# Taking Advantage of Bacterial Adaptation in Order to Optimize Industrial Production of Dry *Propionibacterium freudenreichii*

**DOI:** 10.3390/microorganisms7100477

**Published:** 2019-10-22

**Authors:** Floriane Gaucher, Valérie Gagnaire, Houem Rabah, Marie-Bernadette Maillard, Sylvie Bonnassie, Sandrine Pottier, Pierre Marchand, Gwénaël Jan, Philippe Blanc, Romain Jeantet

**Affiliations:** 1 UMR STLO, Agrocampus Ouest, INRA, F-35042 Rennes, France; floriane.gaucher@inra.fr (F.G.); valerie.gagnaire@inra.fr (V.G.); marie-bernadette.maillard@inra.fr (M.-B.M.); sylvie.bonnassie@univ-rennes1.fr (S.B.); romain.jeantet@agrocampus-ouest.fr (R.J.); 2 Bioprox, 6 rue Barbès, 92532 Levallois-Perret, France; p.marchand@bioprox.com (P.M.); p.blanc@bioprox.com (P.B.); 3 Bba, Pôle Agronomique Ouest, Régions Bretagne et Pays de la Loire, F-35042 Rennes, France; 4 Université de Rennes I, University Rennes, 35000 Rennes, France; 5 University Rennes, CNRS, ISCR-UMR 6226, PRISM, BIOSIT-UMS 3480, F-35000 Rennes, France; sandrine.pottier@univ-rennes1.fr

**Keywords:** osmoadaptation, propionibacteria, stress tolerance, industrialization, probiotic, spray drying

## Abstract

*Propionibacterium freudenreichii* is a beneficial bacterium, used both as a probiotic and as a cheese starter. Large-scale production of *P. freudenreichii* is required to meet growing consumers’ demand. Production, drying and storage must be optimized, in order to guarantee high *P.* *freudenreichii* viability within powders. Compared to freeze-drying, spray drying constitutes the most productive and efficient, yet the most stressful process, imposing severe oxidative and thermal constraints. The aim of our study was to provide the tools in order to optimize the industrial production of dry *P.* *freudenreichii*. Bacterial adaptation is a well-known protective mechanism and may be used to improve bacterial tolerance towards technological stresses. However, the choice of bacterial adaptation type must consider industrial constraints. In this study, we combined (i) modulation of the growth medium composition, (ii) heat-adaptation, and (iii) osmoadaptation, in order to increase *P.* *freudenreichii* tolerance towards technological stresses, including thermal and oxidative constraints, using an experimental design. We further investigated optimal growth and adaptation conditions, by monitoring intracellular compatible solutes accumulation. Glucose addition, coupled to heat-adaptation, triggered accumulation of trehalose and of glycine betaine, which further provided high tolerance towards spray drying and storage. This work opens new perspectives for high quality and fast production of live propionibacteria at the industrial scale.

## 1. Introduction

Diet plays a pivotal role in the maintenance of physiology and of health, with a prominent effect on shaping the structure and activity of the gut microbiota. Dysbiosis, i.e., rupture of the gut microbiota homeostasis, is involved in inflammatory, allergic and atopic disorders [1,2,3]. Over the past decades, the diet of European countries evolved due to changes in life style, and this coincided with an increase in the incidence of inflammatory bowel disease (IBD) and of irritable bowel syndrome (IBS). This constitutes a public health concern [4,5], and to improve health, five European countries advise to consume yogurt or probiotics in their dietary guidelines [6]. Probiotics are defined as “live microorganisms which, when administered in adequate amounts confer a health benefit on the host” [7]. Different species of *Lactobacillus*, *Bifidobacterium*, *Enterococcus*, *Propionibacterium*, *Lactococcus*, *Streptococcus,* and *Saccharomyces* have been investigated for their probiotic properties [8]. Probiotics can exert positive effects on gut inflammatory disorders. As an example, a mixture of probiotic bacteria induced or prolonged remissions in patients suffering from ulcerative colitis [9]. The exposition to key immunomodulatory probiotic or symbiotic bacteria can prevent atopy and allergy [10,11]. Early colonization of the gut by such bacteria decrease the susceptibility to necrotizing enterocolitis in preterm infants with a dysbiotic microbiota [12].

Propionibacteria and bifidobacteria were identified as protective and early colonizing probiotic symbionts [12,13]. Breast-feeding, or feeding infant formula containing such probiotic bacteria, was further shown to prevent dysbiosis-related diseases [14,15]. The gut microbiota can be modulated by the consumption of selected strains of *P. freudenreichii* [16,17,18,19]. Moreover, some strains of this species can modulate the mucosal immune response by inducing anti-inflammatory cytokines in epithelial and immune human cells [20,21]. Propionibacteria is Generally Recognized-As Safe (GRAS) and possesses the Qualified Presumption of Safety (QPS) [22]. This bacteria is used during Swiss-cheese manufacturing and for the production of nutritional and antimicrobial molecules [23]. During fermentation, *Propionibacterium. freudenreichii* produces the beneficial short chain fatty acids propionate and acetate as final products. It also releases the B9 (folate) and B12 (cobalamin) vitamins, together with bifidogenic compounds: DHNA (1,4-dihydroxy-2-naphtoic acid) and ACNQ (2-amino-3-carboxy-1,4-naphthoquinone). DHNA and ACNQ are thought to be responsible for gut microbiota beneficial modulation [23].

*P. freudenreichii* should be consumed alive, for an optimal beneficial effect. Starters and probiotics are generally produced as a powder form. Powders allow extended shelf-life and facilitate storage transport of bacteria. Spray drying is the most efficient drying process, with a lower energy consumption and a higher productivity than freeze-drying [24]. It is furthermore a continuous and fast process, as water evaporation is accelerated by an increase of air/product contact surface by spraying in fine droplets. However, bacteria still suffer from heat and oxidative stresses during the spray drying process, which still needs optimization [24].

The ability of bacteria to adapt is usually harnessed to induce homo- or hetero-protections, called cross-protections. Osmoadaptation is well known to increase *P. freudenreichii* tolerance towards heat, acid, and bile salt stresses [25,26,27]. Accordingly, growth in hyperosmotic conditions increases *Lactobacillus paracasei* and *P. freudenreichii* viability during spray drying [26,28]. During osmoadaptation, bacteria accumulate compatible solutes, which maintain turgescent pressure and enable cell growth and division. Compatible solutes may be imported from the growth medium or synthesized de novo [29]. As an example, *P. freudenreichii* is able to accumulate glycine betaine, glutamate and trehalose for osmoadaptative purposes. Lactose present in hyper-concentrated sweet whey leads to trehalose accumulation and to higher *P. freudenreichii* viability during heat challenge and during spray drying [26]. Addition of salt in the medium decreases significantly the growth rate [30], posing a problem to industries during starter and probiotic production. Lactose addition into the growth medium is also critical, as some consumers reject its utilization because of their lactose intolerance.

In this study, the osmoadaptation was induced at the end of the stationary phase to avoid reduced growth. Glucose, a food-grade carbohydrate much more available and affordable than, for example, trehalose, was chosen. Heat adaptation has been reported to increase *Lactobacillus paracasei* viability during spray drying [28] and *Lactobacillus plantarum* viability during storage [31]. In this work, we thus cultivated *P. freudenreichii* in growth medium with different concentrations of added glucose, we triggered heat and/or osmotic adaptation(s) after the beginning of the stationary phase, and we sought induced protection towards spray drying and storage. We optimized the conditions of *P. freudenreichii* growth and adaptation with an experimental design, in order to improve cell viability upon spray drying. We quantified compatible solutes to better understand *P. freudenreichii* cross-protections. We further checked the starter capacities of *P. freudenreichii* after adaptations and drying. This work opens new avenues for the optimization of the industrial production of dry *P. freudenreichii*.

## 2. Materials and Methods

### 2.1. Strains and Pre-Culture

*Propionibacterium freudenreichii* CIRM-BIA 129 (equivalent to ITG P20) was provided by CNIEL. *Lactobacillus delbrueckii* CIRM-BIA 209 and *Streptococcus thermophilus* CIRM-BIA 67 were provided by the CIRM-BIA Biological Resource Center (Centre International de Ressources Microbienne—Bactéries d’Intérêt Alimentaire, CIRM-BIA, INRA, Rennes, France). *P. freudenreichii, L. delbrueckii,* and *S. thermophilus* were stored, maintained and routinely cultivated in Yeast Extract Lactate (YEL) medium at 30 °C, in Man Rogosa and Sharp (MRS) medium at 37 °C and brain heart infusion medium (BHI) at 43 °C respectively without agitation [32,33].

### 2.2. Optimization of P. freudenreichii Resistance to Heat and Oxidative Challenges

#### 2.2.1. Growth and Adaptation Conditions in the Experimental Design

The JMP Software from SAS was used to study the impact of glucose addition, heat-adaptation, and osmoadaptation on *P. freudenreichii* resistance to heat and oxidative stress. More precisely, glucose was added in YEL medium with concentrations ranging from 0 to 30 g·L^−1^. *P. freudenreichii* was grown at 30 °C over 32 h to 46 h with gentle agitations (40 rpm) to decrease the growth rate [34,35]. At the beginning of the stationary phase, NaCl was added to cultures at a concentration ranging from 0 to 0.9 M during 2 h. Then, the cultures were heated at 42 °C during 0, 1, or 2 h. Adaptations were defined by the experimental design (Table 1).

#### 2.2.2. Stress Challenges

Heat and oxidative challenges were applied to cultures after adaptations as previously described [25]. Heat challenge was performed by placing 2 mL (in a 15 mL Falcon tube) of *P. freudenreichii* cultures in a water bath at 60 °C over 10 min. Oxidative challenge was performed by adding 1.25 mM of hydrogen peroxide (Labogros, France) to 2 mL of culture during 1 h at 30 °C. CFU counting was made on untreated bacteria and after challenges in order to calculate survival percentage. The samples tested are reported in Table 1 and tests were performed in duplicate.

Statistics analyses were done to describe the effect of glucose concentration, heat adaptation and osmoadaptation on *P. freudenreichii* viability during heat and oxidative challenges. The software JUMP was used to fit the second order model to the independent variable. Only variables with a significance higher than 95% (*p* < 0.05) were included in final models. Surface response were drawn to illustrate the main and interactive effects of the independent variables on survival to heat and oxidative challenges. These challenges simulated the stressful spray drying process.

### 2.3. Identification and Quantification of Compatible Solutes Accumulated by P. freudenreichii CIRM−BIA 129

#### 2.3.1. Extraction of Accumulated Compatible Solutes

*P. freudenreichii* CIRM-BIA 129 was cultivated in YEL medium, YEL medium containing 15 g·L^−1^ glucose added with heat adaptation of 1 h at 42 °C (YEL + 15 g glucose+ 42 °C, 1 h) and YEL with 30 g·L^−1^ glucose added with an heat adaptation of 2 h at 42 °C (YEL + 30 g Glucose+ 42 °C, 2 h). Cells were then harvested by centrifugation (8000 *g* 10 min) and were washed twice in a NaCl solution with the same osmolality than the culture medium. Cells were then re-suspended in 2 mL of distilled water, then 8 mL of absolute ethanol were added. The suspension was homogenized and centrifuged (8000 *g*, 10 min) in order to remove cell fragments. The supernatant extract was evaporated over 7 h with a rotary evaporator. Dried extracts were then solubilized in deuterium oxide (Sigma-Aldrich, Saint Louis, MO, USA) as previously described [25].

#### 2.3.2. NMR (Nuclear Magnetic Resonance) Analyses

All ^1^H and ^13^C NMR spectra were recorded at 298 K on a Bruker Advance 500 spectrometer equipped with a 5 mm TCI triple-resonance cryoprobe (PRISM core facility, Rennes, France). ^1^H spectra were acquired with a 6 kHz spectral width, 32 K data points and a total repetition time of 6.73 s. ^13^C spectra were acquired using a proton power-gated decoupling sequence with a 30° flip angle, a 30 kHz spectral width, 64 K data points and a total repetition time of 3.08 s. The data were processed with Topspin software (Bruker Biospin, Wissembourg, France). Before applying the Fourier transform, free induction decays of ^1^H spectra were treated with an exponential broadening of 0.3 Hz.

Samples were solubilized in D_2_O. 3-(trimethylsilyl) propionic-2,2,3,3-d4 acid sodium salt (TSP-d4) (Sigma-Aldrich, Saint Louis, MO, USA) served as an internal reference for ^1^H and ^13^C chemical shifts.

The relative concentration of trehalose, glutamate, proline, and glycine betaine in the samples was determined by integration of their ^1^H signals. Results are expressed as NMR relative units (RU) reported to one unit of optical density (equivalent to 1 mL of culture at an OD of 1).

### 2.4. Spray Drying and Powder Storage

*P. freudenreichii* was cultivated as previously either in YEL medium, or in YEL medium containing glucose, with 1 or 2 h of heat adaptation at 42 °C. (YEL + 15 g glucose+ 42 °C, 1 h and YEL + 30 g Glucose+ 42 °C, 2 h). 20% (*w/w*) of maltodextrine (DE = 6–8) was added to the cultures at the beginning of the stationary phase or after heat adaptation. These different mixtures (∼2 L) were shaken for 10 min prior to delivery to the dryer by a peristaltic pump (520S, Watson-Marlow, France).

A two-fluid nozzle with a diameter of 0.8 mm was used for atomization. The inlet air temperature was fixed at 160 °C. The temperature outlet air was controlled at 60 ± 2 °C, by adjusting the feed rate. The bacterial viabilities were estimated by numeration on YEL agar plates before and after spray drying as described previously [36]. The powders were collected and sealed in sterilized polystyrene bottles (Gosselin, France), stored at a controlled temperature of 4, 20 or 37  °C, and kept away from light over 145 days. One gram of powder was solubilized in 9 g of sterile water and bacterial viability was tested by numeration on YEL agar plates incubated at 30 °C.

### 2.5. Random Amplified Polymorphic DNA (RAPD) Analysis

RAPD was performed using two RAPD primers (primer 1: AAGAGCCCGT and primer 2: AACGCGCAAC) as previously described [37,38]. The PCR mix (2 µL) consisted of Taq buffer with MgCl2 (Q-Biogene/EPTQA100), dNTPs (2 mM/µL, Q-Biogene/NTPMX050), random primer 100 mM/µL, RAPD Analysis Kit GE Healthcare), Taq DNA polymerase (2.5 U/µL), 1 µL of the extracted DNA (25 ngL/µL) and sterile water QSP 25 µL. DNA amplification was performed in a C10000TM thermal cycler (Bio-Rad) using the following conditions: 94 °C for 2 min; 40 cycles of 94 °C for 1 min, 42 °C for 20 s and 72 °C for 2 min; and a final extension step at 72 °C for 10 min. The amplified products were resolved by electrophoresis (100 V, 1 h) on 1% agarose gel (*w/v*) in 0.5 × TBE buffer.

### 2.6. Revivification of dried P. freudenreichii in a cheese-like dairy medium

#### 2.6.1. Culture with Dried *P. freudenreichii* in a Cheese-Like Dairy Medium

Cow milk ultrafiltrate was prepared by milk ultrafiltration (cut-off 8 kDa) as previously described [37]. The two major lactic acid starters, *Lactobacillus delbrueckii* CIRM-BIA 209 and *Streptococcus thermophilus* CIRM-BIA 67, were co-cultivated at 43 °C over 12 h in milk ultrafiltrate containing 5 g·L^−1^ of casein peptone. Cultures were centrifuged in order to eliminate lactic acid bacteria. The supernatants were collected, the pHs were readjusted to 7 using a solution of NaOH 5 M, then the supernatants were filter-sterilized (Top filter PES, 0.45 µm, Nalgene Company, NY, USA). Then, 1.5 g of *P. freudenreichii* powder dried previously was inoculated in 400 mL of this preconditioned milk ultrafiltrate (named later cheese-like medium). A control was performed using a fresh liquid *P. freudenreichii* culture realized in the cheese-like medium supplemented by 1.5 g of maltodextrine. Cultures were incubated at 24 °C over 14 days in order to mimic growth conditions in the warm room during Emmental cheese making [38]. Growth curves were established by CFU numeration on YEL agar plates.

#### 2.6.2. Organic Acids Quantification

Supernatants of the different cultures were harvested by centrifugation during the exponential phase, the beginning of the stationary phase and after 14 days of growth. Culture supernatants were half-diluted with 0.02 M H_2_SO_4_ and centrifuged at 8000 *g* during 30 min at 4 °C to discard the protein pellet prior to filter-sterilization and analysis. Organic acids and sugar were separated using a Minex A-6 ion exchange column (Dionex, Sunnyvale, CA, USA) at 55 °C with 0.01 M H_2_SO_4_ as eluent at a flow rate of 1 mL min^−1^. Both UV (210 nm) and refractometric detectors were used. Appropriate standards of lactose, acetate, propionate, and lactate were used as described previously [39].

### 2.7. Statistical Analysis

The data were from triplicate samples. All the results are presented as mean values with standard deviation. Statistical significance was set at *p* < 0.05. Calculations were performed using GraphPad Prism Software (Prism 7 for Windows, Redmond, WA, USA).

## 3. Results

### 3.1. Optimization of P. freudenreichii Stress Tolerance with an Experimental Design

**Figure 1:**
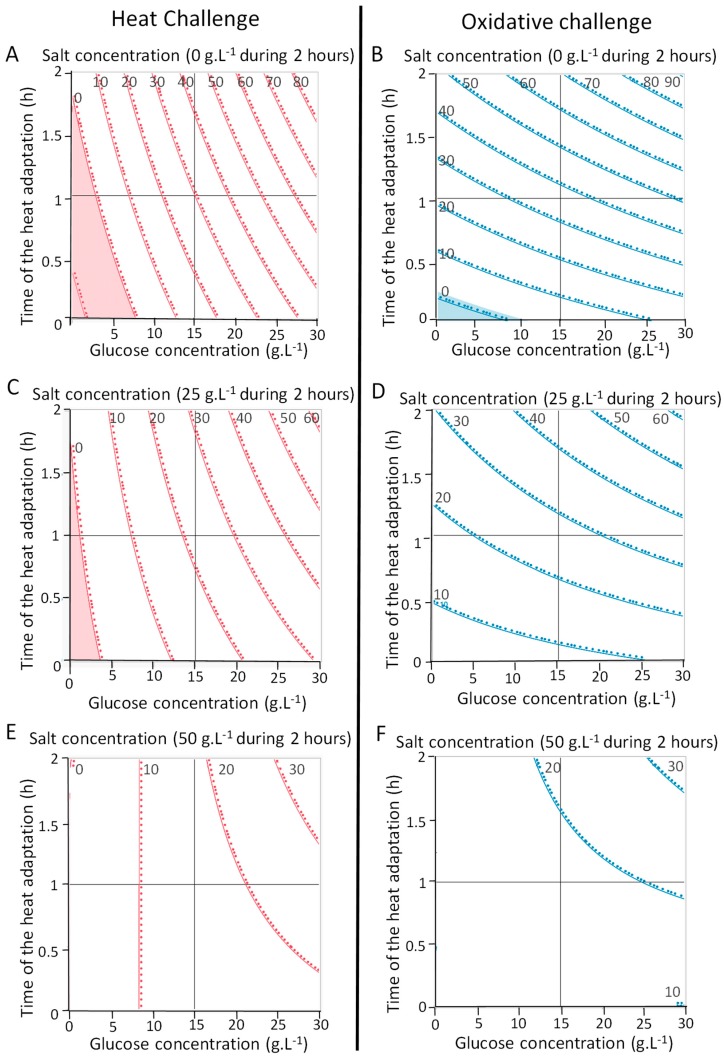
Adaptations and growth medium compositions modulate *P. freudenreichii* stress tolerance. Effect of addition of glucose, heat-adaptation and osmoadaptation were tested on *P. freudenreichii* CIRM-BIA 129 stress tolerance following an experimental design. *P. freudenreichii* was cultivated in YEL medium with 0.15 or 30 g·L^−1^ of glucose until the beginning of the stationary phase. Cultures were then subjected to osmoadaptation (0.25 or 50 g·L^−1^ of NaCl) over 2 h and then heat-adapted at 42 °C over 0, 1 or 2 h. Cultures were subjected to heat challenge (**A**,**C**,**E**, 60 °C for 10 min) or oxidative challenge (**B**,**D**,**F**, 1.15 mM H_2_O_2_ for 1 h) as described in materials and methods. *P. freudenreichii* viability after challenges was determined by CFU counting before and after challenges. Results are expressed as percent survival and reported near the iso-response curves.

We analyzed *P. freudenreichii* survival rates upon heat and oxidative challenges in order to estimate individual and interactive coefficients effect on *P. freudenreichii* tolerance towards heat challenge (Table 2) and oxidative challenge (Table 3). Salt addition after the beginning of the stationary phase had a negative impact on *P. freudenreichii* survival during heat and oxidative challenges (Figure 1) (coefficient estimated: –7% and –10%, in Table 2 and Table 3, respectively), whereas glucose addition and heat adaptation had a positive impact on *P. freudenreichii* survival during these challenges (Figure 1). Indeed, glucose addition increased *P. freudenreichii* viability during heat challenge (coefficient estimated: +24%) more than heat adaptation (coefficient estimated: +9%). During oxidative challenge, heat-adaptation had a higher impact than glucose addition (coefficient estimated: +19% and +10%, respectively). Best survival during both challenges was obtained when *P. freudenreichii* was grown in the presence of 30 g·L^−1^ of glucose and heat-adapted (42 °C, 2 h) at the beginning of stationary phase (Figure 1).

### 3.2. P. freudenreichii Viability after Spray Drying and Storage

**Figure 2:**
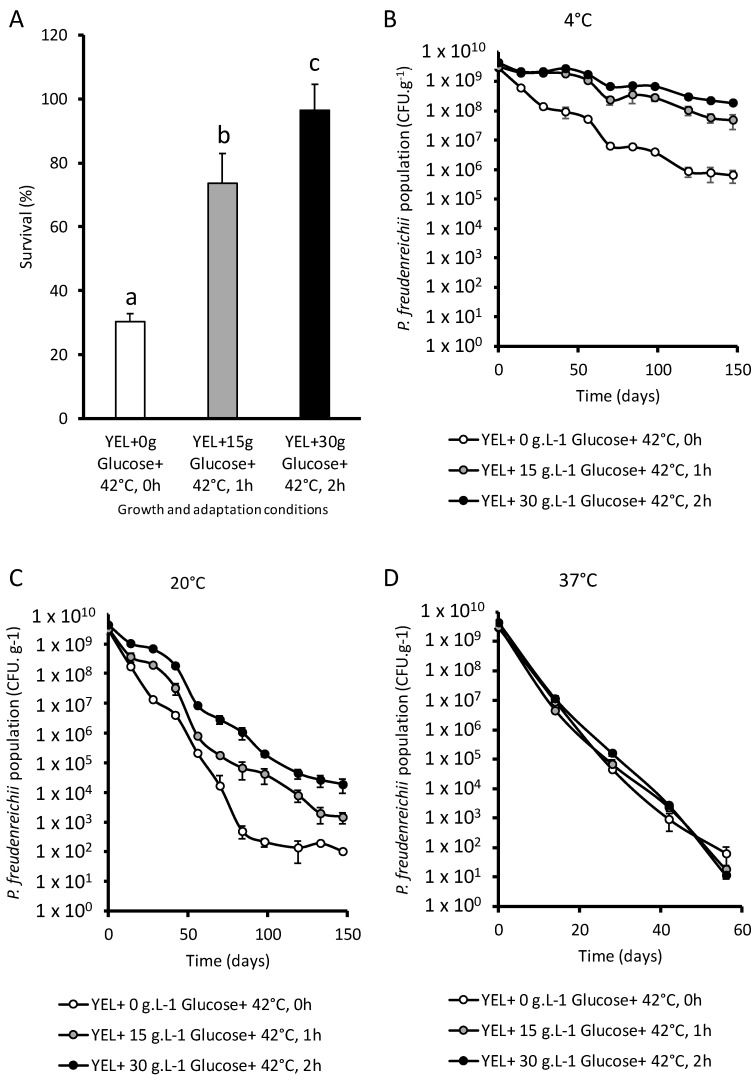
Glucose addition in growth medium and heat-adaptation improve *P. freudenreichii* viability during spray drying and storage. *P. freudenreichii* CIRM-BIA 129 were cultivated in YEL (YEL+ 0 g Glucose + 42 °C, 0 h), in YEL medium containing 15 g·L^−1^ of glucose and heat-adapted at 42 °C over 1 h (YEL + 15 g Glucose + 42 °C, 1 h) or YEL medium containing 30 g·L^−1^ of glucose and heat-adapted at 42 °C over 2 h (YEL + 15 g Glucose + 42 °C, 2 h) until the beginning of the stationary phase. Maltodextrine was added in the cultures with a final concentration of 20% (*w/w*) to increase the dry extract. Cultures were then spray dried (**A**) and *P. freudenreichii* survival was quantified by CFU counting as described in materials and methods. Results are expressed as percent survival. The different powders obtained were stored at 4 °C (**B**), 20 °C (**C**) and 37 °C (**D**) over 145 days. *P. freudenreichii* viability was quantified by CFU counting during storage. Error bars represent the standard deviation for triplicate experiments. Significant differences are reported with different letters above the columns (*p* > 0.05).

**Figure 3:**
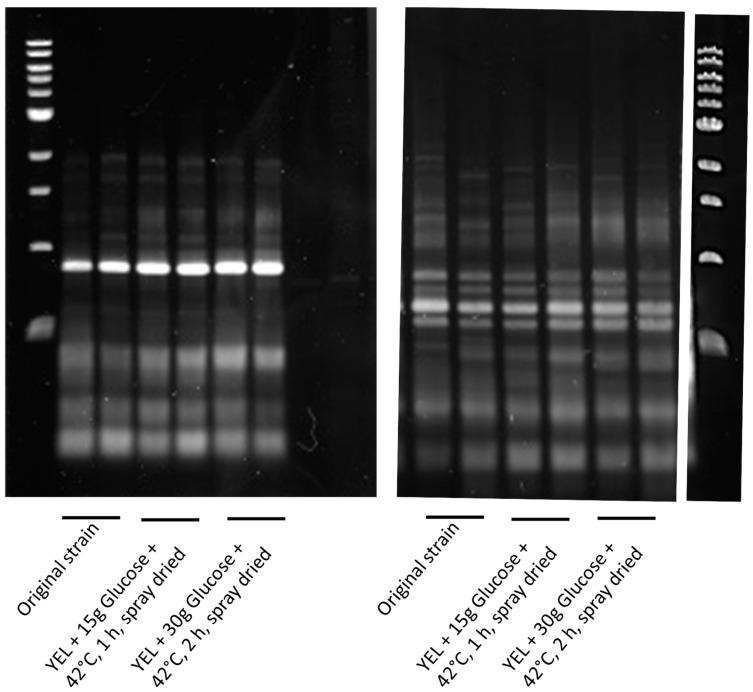
RAPD analysis of *P. freudenreichii* cultures before and after adaptation and drying *P. freudenreichii*. Colonies were isolated from the original culture and from powders after adaptation and spray drying. RAPD was performed using two RAPD primers (left: primer 1: AAGAGCCCGT and right: primer 2: AACGCGCAAC) as described in “Materials and Methods”. Amplified products were resolved by electrophoresis (100 V, 1 h) on 1% agarose gels. First and last lines correspond to 1 kb DNA ladder.

Control cultures (YEL), in the best conditions defined by the experimental design (YEL + 30 g glucose + 42 °C, 2 h) and in the intermediate tolerance conditions (YEL + 15 g glucose + 42 °C, 1 h) were spray dried. *P. freudenreichii* grown in YEL with 15 g of glucose and heat-adapted during 1 h at 42 °C survived better to spray drying than unadapted cells (74% and 30% survival, respectively) (Figure 2A). A higher glucose concentration in the culture medium with a longer thermal adaptation further increased the survival during spray drying (96.4%). The addition of glucose and/or the thermal adaptation had a positive impact on *P. freudenreichii* viability during spray drying. During storage at 4 and 20 °C, *P. freudenreichii* grown with glucose and then heat-adapted displayed enhanced viability (Figure 2B,C). The highest the glucose concentration and the longest the heat-adaptation, the lowest *P. freudenreichii* death occurred during storage at 4 and 20 °C. During storage at 37 °C, these adaptations had no impact on cells’ death (Figure 2D). As a quality control, *P. freudenreichii* colonies were isolated before and after adaptation and spray drying, prior to RAPD analysis. Figure 3 shows that RAPD profiles were identical in the three conditions; original culture, spray dried after adaptation (YEL + 15 g glucose + 42 °C, 1 h), and spray dried after (YEL + 30 g glucose + 42 °C, 2 h).

### 3.3. Compatible Solutes Accumulation Depends on P. freudenreichii Growth Medium and Adaptation Conditions

**Figure 4:**
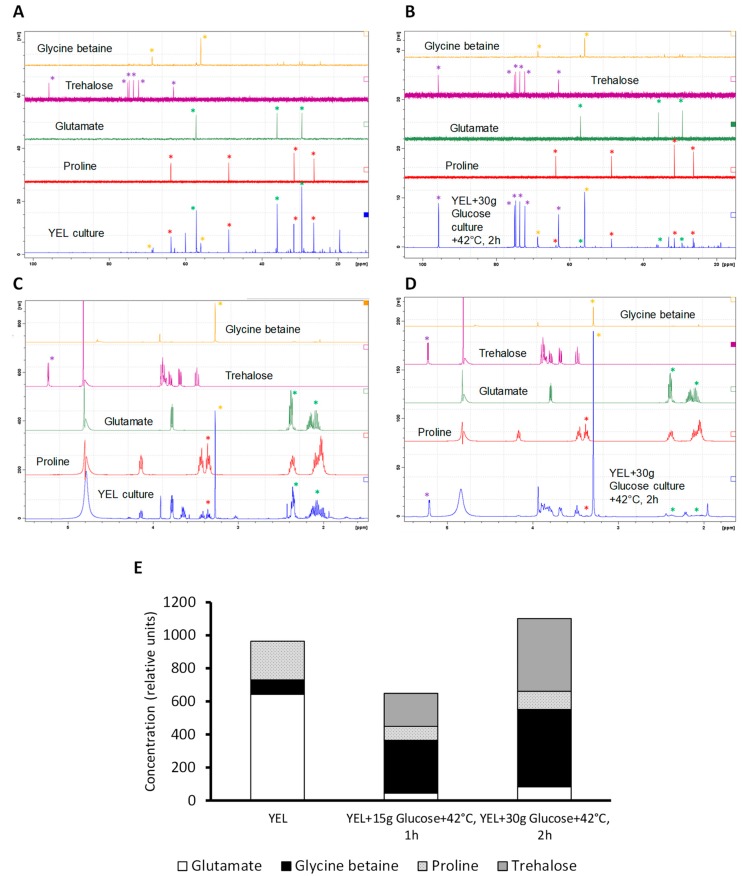
*P. freudenreichii* compatible solutes is modulated by adaptations. *P. freudenreichii* CIRM-BIA 129 were cultivated in YEL (YEL + 0 g Glucose + 42 °C, 0 h), in YEL medium containing 15 g·L^−1^ of glucose and heat-adapted at 42 °C over 1 h (YEL + 15 g Glucose + 42 °C, 1 h) or YEL medium containing 30 g·L^−1^ of glucose and heat-adapted at 42 °C over2 h (YEL + 30 g Glucose + 42 °C, 2 h) until the beginning of the stationary phase. Cytoplasmic extract was made as described in materials and methods. The signals used for the identification (**A**,**B**, C-NMR) and for the quantification (**C**,**D**, H-NMR) of the solutes are shown by colored asterisks. The spectra shown correspond to glycine betaine (yellow), trehalose (purple), glutamate (green), proline (red) and cytoplasmic hydroalcoholic extract (blue). The examples shown corresponds to the control growth conditions (YEL culture, **A**,**C**) and to adapted conditions (YEL + 30 g Glucose + 42 °C, 2 h, **B**,**D**). Spectra corresponding to all conditions are illustrated in the Supplemental Figure. Compatible solutes accumulation is expressed in relative units (**E**).

We used NMR as the standard used in microbial physiology for evidence accumulation of compatible solutes as a result of osmoadaptation [26,40,41,42]. The accumulated compatible solutes were analyzed by NMR as described [43,44,45,46]. As shown in Figure 4A–D), the spectra corresponding to glycine betaine (yellow), trehalose (purple), glutamate (green), and proline (red) allowed us to identify and quantify these compounds in cytoplasmic hydroalcoholic extract (blue). *P. freudenreichii* CIRM-BIA 129 was able to accumulate glycine betaine, trehalose, glutamate, and proline during growth and adaptations (Figure 4E). In the control medium (YEL), *P. freudenreichii* accumulated high amounts of glutamate (66.7%), and to a lesser extent proline (24.2%) and little amounts of glycine betaine (9.1%). This medium is isotonic with an osmolality of 0.308 osmol. The addition of 15 g of glucose to this culture medium (0.433 osmol), as well as heat-adaptation over 1 h, drastically affected compatible solutes accumulation. First, they decreased the total amount of compatible solute accumulated. Second, they changed the proportions of compatible solutes accumulated. Compared to the control medium, glycine betaine and trehalose were accumulated in higher amount (49.4% and 306 % respectively), with lower amounts of proline (13.0%) and glutamate (7.0%). Higher concentration of glucose (30 g·L^−1^: 0.455 osmol) in the YEL medium and a longer heat- adaptation (2 h) further increased the amount of compatible solutes accumulated by *P. freudenreichii*, but with a profile similar to that of *P. freudenreichii* grown with 15 g of glucose and heat-adapted for 1 h. Indeed, *P. freudenreichii* accumulated in this case trehalose (40%) and glycine betaine (42.5%) in high amounts, with lower amounts of proline (10%) and of glutamate (10%).

### 3.4. P. freudenreichii Revivification

#### 3.4.1. Growth of Dried *P. freudenreichii* in a Cheese-Like Medium

**Figure 5:**
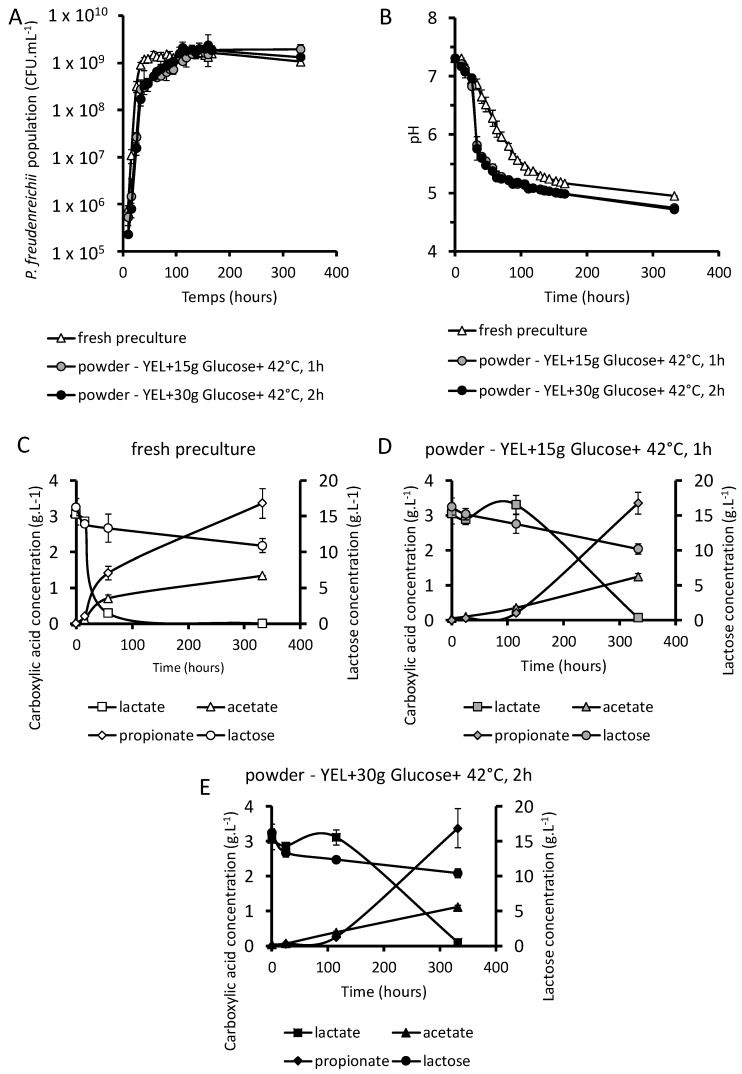
Powders containing *P. freudenreichii* were used to ferment a cheese-like medium. Powders obtained previously were inoculated in a cheese-like medium. *P. freudenreichii* growth was monitored by CFU counting in YEL agar plates (**A**) and acidification were followed (**B**). Lactate and lactose consumption, as well as propionate and acetate production, were quantified by HPLC (**C**–**E**) as described in material and methods. Fresh culture: inoculation with *P. freudenreichii* grown in YEL medium. (YEL + 15 g Glucose + 42 °C, 1 h): inoculation with powders containing *P. freudenreichii* cultivated in YEL with 15 g·L^−1^ of glucose and heat adapted at 42 °C over 1 h before spray drying. (YEL + 15 g Glucose + 42 °C, 1 h): inoculation with powders containing *P. freudenreichii* cultivated in YEL with 15 g·L^−1^ of glucose and heat adapted at 42 °C over 1 h before spray drying.

Powders containing *P. freudenreichii*, or a fresh culture (*P. freudenreichii* in stationary phase) in YEL medium as a control, were inoculated in a cheese-like medium consisting of milk ultrafiltrate previously fermented by a mixture of *L. delbrueckii* and a *S. thermophilus*, the major lactic starters implemented together with *P. freudenreichii* in Emmental cheese. In the absence of adaptation, *P. freudenreichii* was not able to grow in the cheese-like medium and its population remained constant throughout the time of incubation in this medium. Conversely, adapted cultures and dried *P. freudenreichii* powders were able to grow in the cheese-like medium (Figure 5A), indicating revivification of the propionic starter, so that it reached final propionibacterial populations equal to the control. At the end of the fermentation, all cultures had a pH between 4.72 and 4.95, but the pH decreased more slowly with the fresh culture, when compared to dried propionibacteria (Figure 5B).

#### 3.4.2. Fate of Saccharides and Organic Acids

Utilization of lactose and of lactate, as well as production of propionate and of acetate, the main end products of propionic fermentation, were monitored as described in materials and methods. Lactose utilization was nearly the same between dried *P. freudenreichii* and the fresh culture (Figure 5C–E). Dried *P. freudenreichii* consumed lactate and produced propionate and acetate less rapidly than the fresh culture. Final consummation of lactate and lactose and final production of acetate and propionate were non-significantly different between fresh preculture and dried *P. freudenreichii*. Propionate final concentration was twice the acetate final concentration. Interestingly, in the case of dried propionibacteria, lactose was consumed as soon as the beginning of growth, while lactate remained first constant before being consumed after 100 h of growth. In the case of fresh precultures, both lactose and lactate were used throughout the culture.

## 4. Discussion

### 4.1. Addition of Glucose and Heat-Adaptation Both Induce Cross-Protections to P. freudenreichii

Glucose addition and heat adaptation both improved *P. freudenreichii* tolerance towards oxidative and heat challenges (Figure 1). Heat adaptation haas already been described to increase *P. freudenreichii, Enterococcus faecalis,* and *Escherichia coli* thermotolerance [44,47,48]. Addition of glucose during growth also improved *P. freudenreichii* viability during heat and oxidative stress. Accordingly, growth in hyper-concentrated sweet whey, a medium rich in the saccharide lactose, was previously shown to induce such cross-protection, in parallel with trehalose accumulation [26,36]. We showed here that addition of the monosaccharide glucose in combination with a thermo-adaptation also triggers cross-protections and the accumulation of trehalose and of glycine betaine. Glucose is known to be a trehalose building block in a wide range of microorganisms [49]. The experimental design highlighted the positive effect of interactions between the addition of glucose and the heat adaptation. The condition allowing the best survival during oxidative and heat challenges was the YEL medium containing 30 g·L^−1^ of glucose and heat-adaptation during 2 h at 42 °C (Figure 1). Following glucose addition and thermal adaptation, we also tested addition of NaCl after growth and observed afterwards a reduced tolerance. Growth in the presence of NaCl may induce tolerance [25,44,50], while NaCl addition after growth does not.

### 4.2. Combining Glucose Aaddition and Thermo-Adaptation Increases P. freudenreichii Viability during Spray Drying and Storage

Glucose addition in the growth medium and heat adaptation at the beginning of the stationary phase increased *P. freudenreichii* survival after drying (Figure 2A). Increasing the glucose concentration and/or the duration of heat adaptation led to higher survival as predicted by the experimental design. Heat adaptation has already been reported to improve *Lactobacillus paracasei* and *Candida sake* survival during spray drying [28,51]. Over storage at 4 and 20 °C, dried *P. freudenreichii* grown with glucose and heat-adapted exhibited a better survival rate than the control (Figure 2B,C). In other words, a higher glucose concentration and a longer heat-adaptation increased survival during storage as during spray-drying. Similarly, heat adaptation was reported to increase *L. plantarum* viability during storage [31]. Sucrose addition can increase *Lactobacillus sakei* survival during storage after spray drying [52]. However, the impact of saccharides on bacteria viability during storage is not well known yet.

### 4.3. Combining Glucose Addition and Thermo-Adaptation Leads to Compatible Solutes Accumulation and Increases P. freudenreichii Viability to Spray Drying

Control cultures accumulated a high amount of the compatible solutes glutamate and proline. This can be explained by the culture agitation and the presence of oxygen. Indeed, such accumulation was not observed in the same YEL medium in the absence of agitation (data not shown). In this study, proline was accumulated under the three different conditions. This amino acid is accumulated by *Brevibacterium flavum* in the presence of dissolved oxygen and is biosynthesized from glutamate [53]. In the presence of glucose and under heat-adaptation, *P. freudenreichii* accumulated high amounts of glycine betaine and of trehalose. Glycine betaine is known to play a role during thermo-protection [54,55]. Trehalose accumulation is well known during heat-adaptation in yeast [56,57], and *P. freudenreichii* is known to accumulate trehalose [41]. Increased glucose concentration in the growth medium led to increased trehalose accumulation, as described previously [41]. Lactose reportedly leads to higher trehalose accumulation by *P. freudenreichii* than glucose [26,41]. Addition of glucose coupled to heat adaptation increase trehalose accumulation by *Saccharomyces cerevisiae* [57]. When *P. freudenreichii* was grown with high glucose concentration and was heat-adapted, its trehalose accumulation and its resistance to heat challenges increased. Accordingly, trehalose accumulation is determinant for thermo-tolerance for *S. cerevisiae* and *P. freudenreichii* [26,58].

### 4.4. Combining Glucose Addition and Heat Adaptation Leads to Production of Efficient Propionic Starter

*P. freudenreichii*, in the cheese-like medium, exhibited similar growth parameters, whether inoculated as a fresh culture, or as a powder obtained from an adapted culture. Compared to the control fresh culture, dried *P. freudenreichii* acidified more and faster the cheese-like dairy medium. Lactate concentration decreased later in cultures inoculated with powders containing adapted *P. freudenreichii,* which delayed the production of propionate and acetate. In all cultures, *P. freudenreichii* consumed both lactate and lactose and produced acetate and propionate with a 2:1 ratio, that is characteristic of propionic fermentation according to the Fitz equation [59]. The amount of lactose, lactate, propionate, and acetate were similar for all cultures at the end of fermentation. However, *P. freudenreichii* used both lactose and lactate as soon as the beginning of growth in the case of fresh cultures. By contrast, *P. freudenreichii* preferentially used lactose at the beginning of growth, in accordance with faster acidification, in the case of dried bacteria. *P. freudenreichii* grown in presence of glucose and heat-adapted displayed enhanced resistance to spray drying and enhanced acidification ability. Such powders containing adapted *P. freudenreichii* can thus be used as efficient cheese starters.

## 5. Conclusions

To conclude, we modulated the growth medium composition and we used thermal adaptation to increase *P. freudenreichii* viability during spray drying and during storage. In the presence of glucose, *P. freudenreichii* accumulated trehalose and glycine betaine, whatever the osmotic pressure. Compared to osmotic adaptation, thermal adaptation is easier to implement in industry, and allows faster production of *P. freudenreichii,* with high viability during spray drying and storage. This work thus opens up new avenues for industrial production of propionic starters.

## Figures and Tables

**Table 1:** Arrangement of the experimental design for the three independent variables used and their level.

	Design Matrice			Working Matrice		
Samples	NaCl Concentration	Glucose Concentration	Time of Heat Adaptation	NaCl Concentration (g·L^−1^)	Glucose Concentration (g·L^−1^)	Time of Heat Adaptation (h)
1	−1	−1	−1	0	0	0
2	−1	−1	0	0	0	1
3	−1	−1	1	0	0	2
4	−1	0	−1	0	15	0
5	−1	0	0	0	15	1
6	−1	0	1	0	15	2
7	−1	1	−1	0	30	0
8	−1	1	0	0	30	1
9	−1	1	1	0	30	2
10	0	−1	−1	25	0	0
11	0	−1	0	25	0	1
12	0	−1	1	25	0	2
13	0	0	−1	25	15	0
14	0	0	0	25	15	1
15	0	0	0	25	15	1
16	0	0	0	25	15	1
17	0	0	1	25	15	2
18	0	1	−1	25	30	0
19	0	1	0	25	30	1
20	0	1	1	25	30	2
21	1	−1	−1	50	0	0
22	1	−1	0	50	0	1
23	1	−1	1	50	0	2
24	1	0	−1	50	15	0
25	1	0	0	50	15	1
26	1	0	1	50	15	2
27	1	1	−1	50	30	0
28	1	1	0	50	30	1
29	1	1	1	50	30	2

**Table 2:** Coefficient estimation of the different variables and their interaction on *P. freudenreichii* viability during heat challenge. NS: not significant.

	Estimation	Prob. >|*t*|
Constant	23	<0.0001
NaCl concentration(0.50)	−7	0.0077
Glucose concentration(0.30)	24	<0.0001
Time of heat adaptation(0.2)	9	0.0011
NaCl concentration × Glucose concentration	−13	0.0003
NaCl concentration × Time of heat adaptation	NS	0.0638
Glucose concentration × Time of heat adaptation	7	0.0429

**Table 3:** Coefficient estimation of the different variables and their interaction on *P. freudenreichii* viability during oxidative challenge. NS: non-significant.

	Estimation	Prob. >|t|
Constant	29	<0.0001
NaCl concentration(0.50)	−10	0.0067
Glucose concentration(0.30)	10	0.0068
Time of heat adaptation(0.2)	19	<0.0001
NaCl concentration × Glucose concentration	NA	0.2329
NaCl concentration × Time of heat adaptation	−14	0.0021
Glucose concentration × Time of heat adaptation	NA	0.1406

## Supplementary Materials

Supplementary materials can be found at https://www.mdpi.com/2076-2607/7/10/477/s1.

## References

[1] Moco S., Candela M., Chuang E., Draper C., Cominetti O., Montoliu I., Barron D., Kussmann M., Brigidi P., Gionchetti P. (2014). Systems biology approaches for inflammatory bowel disease: Emphasis on gut microbial metabolism. Inflamm. Bowel Dis..

[2] Soularue E., Lepage P., Colombel J.F., Coutzac C., Faleck D., Marthey L., Collins M., Chaput N., Robert C., Carbonnel F. (2018). *Enterocolitis* due to immune checkpoint inhibitors: A systematic review. Gut.

[3] Skonieczna-Żydecka K., Marlicz W., Misera A., Koulaouzidis A., Łoniewski I. (2018). Microbiome—the missing link in the Ggt-brain axis: Focus on its role in gastrointestinal and mental health. JCM.

[4] David L.A., Maurice C.F., Carmody R.N., Gootenberg D.B., Button J.E., Wolfe B.E., Ling A.V., Devlin A.S., Varma Y., Fischbach M.A. (2014). Diet rapidly and reproducibly alters the human gut microbiome. Nature.

[5] Plé C., Breton J., Richoux R., Nurdin M., Deutsch S.-M., Falentin H., Hervé C., Chuat V., Lemée R., Maguin E. (2016). Combining selected immunomodulatory *Propionibacterium freudenreichii* and *Lactobacillus delbrueckii* strains: Reverse engineering development of an anti-inflammatory cheese. Mol. Nutr. Food Res..

[6] Ebner S., Smug L.N., Kneifel W., Salminen S.J., Sanders M.E. (2014). Probiotics in dietary guidelines and clinical recommendations outside the European Union. WJG.

[7] FAO/WHO Evaluation of Health and Nutritional Properties of Powder Milk and Live Lactic Acid Bacteria.

[8] Fung W.-Y., Lye H.-S., Lim T.-J., Kuan C.-Y., Liong M.-T., Liong M.-T. (2011). Roles of probiotic on gut health. Probiotics.

[9] Ganji-Arjenaki M., Rafieian-Kopaei M. (2018). Probiotics are a good choice in remission of inflammatory bowel diseases: A meta analysis and systematic review. J. Cell. Physiol..

[10] Butel M.-J., Waligora-Dupriet A.-J., Wydau-Dematteis S. (2018). The developing gut microbiota and its consequences for health. J. Dev. Orig. Health Dis..

[11] Fujimura K.E., Sitarik A.R., Havstad S., Lin D.L., Levan S., Fadrosh D., Panzer A.R., LaMere B., Rackaityte E., Lukacs N.W. (2016). Neonatal gut microbiota associates with childhood multisensitized atopy and T cell differentiation. Nat. Med..

[12] Colliou N., Ge Y., Sahay B., Gong M., Zadeh M., Owen J.L., Neu J., Farmerie W.G., Alonzo F., Liu K. (2017). Commensal *Propionibacterium* strain UF1 mitigates intestinal inflammation via Th17 cell regulation. J. Clin. Investig..

[13] Chang H.-Y., Chen J.-H., Chang J.-H., Lin H.-C., Lin C.-Y., Peng C.-C. (2017). Multiple strains probiotics appear to be the most effective probiotics in the prevention of necrotizing enterocolitis and mortality: An updated meta-analysis. PLoS ONE.

[14] Milani C., Duranti S., Bottacini F., Casey E., Turroni F., Mahony J., Belzer C., Delgado Palacio S., Arboleya Montes S., Mancabelli L. (2017). The first microbial colonizers of the human gut: Composition, activities, and health implications of the infant gut microbiota. Microbiol. Mol. Biol. Rev..

[15] Repa A., Thanhaeuser M., Endress D., Weber M., Kreissl A., Binder C., Berger A., Haiden N. (2015). Probiotics (*Lactobacillus acidophilus* and *Bifidobacterium bifidum*) prevent NEC in VLBW infants fed breast milk but not in formula. Pediatr. Res..

[16] Bouglé N.D., Roland F. (1999). Lebeurrier Effect of Propionibacteria supplementation on fecal Bifidobacteria and segmental colonic transit time in healthy human subjects. Scand. J. Gastroenterol..

[17] Mitsuyama K., Masuda J., Yamasaki H., Kitazaki S., Koga H., Uchida M., Sata M. (2007). Treatment of Ulcerative Colitis with Milk Whey Culture with *Propionibacterium freudenreichii* 2007. J. Intest. Microbiol..

[18] Hojo K., Yoda N., Tsuchita H., Ohtsu T., Seki K., Taketomo N., Murayama T., Iino H. (2002). Effect of ingested culture of *Propionibacterium freudenreichii* ET-3 on fecal microflora and stool frequency in healthy females. Biosci. Microflora.

[19] Seki K., Nakao H., Umino H., Isshiki H., Yoda N., Tachihara R., Ohuchi T., Saruta H., Suzuki K., Mitsuoka T. (2004). Effects of Fermented Milk Whey Containing Novel Bifidogenic Growth Stimulator Produced by *Propionibacterium* on Fecal Bacteria, Putrefactive Metabolite, Defecation Frequency and Fecal Properties in Senile Volunteers Needed Serious Nursing-Care Taking Enteral Nutrition by Tube Feeding 2004. J. Intest. Microbiol..

[20] Rabah H., Ménard O., Gaucher F., do Carmo F.L.R., Dupont D., Jan G. (2018). Cheese matrix protects the immunomodulatory surface protein SlpB of *Propionibacterium freudenreichii* during in vitro digestion. Food Res. Int..

[21] Foligne B., Deutsch S.-M., Breton J., Cousin F.J., Dewulf J., Samson M., Pot B., Jan G. (2010). Promising immunomodulatory effects of selected strains of dairy propionibacteria as evidenced in vitro and in vivo. Appl. Environ. Microbiol..

[22] European Food Safety Authority (EFSA) The Maintenance of the List of QPS Microorganisms Intentionally Added to Food or Feed-Scientific Opinion of the Panel on Biological Hazards 2008. https://www.efsa.europa.eu/fr/efsajournal/pub/923.

[23] Rabah H., Rosa do Carmo F., Jan G. (2017). Dairy propionibacteria: Versatile probiotics. Microorganisms.

[24] Huang S., Vignolles M.-L., Chen X.D., Le Loir Y., Jan G., Schuck P., Jeantet R. (2017). Spray drying of probiotics and other food-grade bacteria: A review. Trends Food Sci. Technol..

[25] Gaucher F., Bonnassie S., Rabah H., Leverrier P., Pottier S., Jardin J., Briard-Bion V., Marchand P., Jeantet R., Blanc P. (2019). Benefits and drawbacks of osmotic adjustment in *Propionibacterium freudenreichii*. J. Proteom..

[26] Huang S., Rabah H., Jardin J., Briard-Bion V., Parayre S., Maillard M.-B., Le Loir Y., Chen X.D., Schuck P., Jeantet R. (2016). Hyperconcentrated sweet whey, a new culture medium that enhances *Propionibacterium freudenreichii* stress tolerance. Appl. Environ. Microbiol..

[27] Jan G., Rouault A., Maubois J.-L. (2000). Acid stress susceptibility and acid adaptation of *Propionibacterium freudenreichii* subsp. shermanii. Lait.

[28] Desmond C., Stanton C., Fitzgerald G.F., Collins K., Paul Ross R. (2001). Environmental adaptation of probiotic lactobacilli towards improvement of performance during spray drying. Int. Dairy J..

[29] Csonka L.N., Hanson A.D. (1991). Prokaryotic Osmoregulation: Genetics and Physiology. Annu. Rev. Microbiol..

[30] Boyaval P., Deborde C., Corre C., Blanco C., Bégué É. (1999). Stress and osmoprotection in propionibacteria. Lait.

[31] Paéz R., Lavari L., Vinderola G., Audero G., Cuatrin A., Zaritzky N., Reinheimer J. (2012). Effect of heat treatment and spray drying on lactobacilli viability and resistance to simulated gastrointestinal digestion. Food Res. Int..

[32] Malik A.C., Reinbold G.W., Vedamuthu E.R. (1968). An evaluation of the taxonomy of *Propionibacterium*. Can. J. Microbiol..

[33] De Man J.C., Rogosa M., Sharpe M.E. (1960). A medium for the cultivation of lactobacilli. J. Appl. Bacteriol..

[34] Demirtas M.U., Kolhatkar A., Kilbane J.J. (2003). Effect of aeration and agitation on growth rate of *Thermus thermophilus* in batch mode. J. Biosci. Bioeng..

[35] Arnaud J.-P., Lacroix C., Choplin L. (1992). Effect of agitation rate on cell release rate and metabolism during continuous fermentation with entrapped growing: *Lactobacillus casei* subsp. casei. Biotechnol. Tech..

[36] Huang S., Cauty C., Dolivet A., Le Loir Y., Chen X.D., Schuck P., Jan G., Jeantet R. (2016). Double use of highly concentrated sweet whey to improve the biomass production and viability of spray-dried probiotic bacteria. J. Funct. Foods.

[37] Cousin F.J., Louesdon S., Maillard M.-B., Parayre S., Falentin H., Deutsch S.-M., Boudry G., Jan G. (2012). The first dairy product exclusively fermented by *Propionibacterium freudenreichii*: A new vector to study probiotic potentialities in vivo. Food Microbiol..

[38] Gagnaire V., Jardin J., Rabah H., Briard-Bion V., Jan G. (2015). Emmental cheese environment enhances *Propionibacterium freudenreichii* stress tolerance. PLoS ONE.

[39] Aburjaile F.F., Rohmer M., Parrinello H., Maillard M.-B., Beaucher E., Henry G., Nicolas A., Madec M.-N., Thierry A., Parayre S. (2016). Adaptation of *Propionibacterium freudenreichii* to long-term survival under gradual nutritional shortage. BMC Genom..

[40] Kets E., Teunissen P., de Bont J. (1996). Effect of compatible solutes on survival of lactic acid bacteria subjected to drying. Appl. Environ. Microbiol..

[41] Cardoso F.S., Castro R.F., Borges N., Santos H. (2007). Biochemical and genetic characterization of the pathways for trehalose metabolism in *Propionibacterium freudenreichii*, and their role in stress response. Microbiology.

[42] Dalmasso M., Aubert J., Even S., Falentin H., Maillard M.-B., Parayre S., Loux V., Tanskanen J., Thierry A. (2012). Accumulation of intracellular glycogen and trehalose by *Propionibacterium freudenreichii* under conditions mimicking cheese ripening in the cold. Appl. Environ. Microbiol..

[43] Behrends V., Bundy J.G., Williams H.D. (2011). Differences in strategies to combat osmotic stress in *Burkholderia cenocepacia* elucidated by NMR-based metabolic profiling: *B. cenocepacia* osmotic stress tolerance. Lett. Appl. Microbiol..

[44] Pleitner A., Zhai Y., Winter R., Ruan L., McMullen L.M., Gänzle M.G. (2012). Compatible solutes contribute to heat resistance and ribosome stability in *Escherichia coli* AW1.7. Biochim. Biophys. Acta (BBA)-Proteins Proteom..

[45] Vaidya S., Dev K., Sourirajan A. (2018). Distinct osmoadaptation strategies in the strict halophilic and halotolerant bacteria isolated from Lunsu salt water body of north west Himalayas. Curr. Microbiol..

[46] Weinisch L., Kirchner I., Grimm M., Kühner S., Pierik A.J., Rosselló-Móra R., Filker S. (2019). Glycine betaine and ectoine are the major compatible solutes used by four different halophilic heterotrophic ciliates. Microb. Ecol..

[47] Leverrier P., Vissers J.P.C., Rouault A., Boyaval P., Jan G. (2004). Mass spectrometry proteomic analysis of stress adaptation reveals both common and distinct response pathways in *Propionibacterium freudenreichii*. Arch. Microbiol..

[48] Flahaut S., Hartke A., Giard J., Benachour A., Boutibonnes P., Auffray Y. (1996). Relationship between stress response towards bile salts, acid and heat treatment in *Enterococcus faecalis*. FEMS Microbiol. Lett..

[49] Streeter J.G., Gomez M.L. (2006). Three enzymes for trehalose synthesis in *Bradyrhizobium* cultured bacteria and in bacteroids from soybean nodules. Appl. Environ. Microbiol..

[50] Teixido N., Canamas T.P., Usall J., Torres R., Magan N., Vinas I. (2005). Accumulation of the compatible solutes, glycine-betaine and ectoine, in osmotic stress adaptation and heat shock cross-protection in the biocontrol agent *Pantoea agglomerans* CPA-2. Lett. Appl. Microbiol..

[51] Cañamás T.P., Viñas I., Usall J., Magan N., Solsona C., Teixidó N. (2008). Impact of mild heat treatments on induction of thermotolerance in the biocontrol yeast *Candida* sake CPA-1 and viability after spray-drying. J. Appl. Microbiol..

[52] Ferreira V., Soares V., Santos C., Silva J., Gibbs P.A., Teixeira P. (2005). Survival of *Lactobacillus sakei* during heating, drying and storage in the dried state when growth has occurred in the presence of sucrose or monosodium glutamate. Biotechnol. Lett..

[53] Akashi K., Shibai H., Hirose Y. (1979). Effect of oxygen supply on l -lysine, l -threonine and l -isoleucine fermentations. Agric. Biol. Chem..

[54] Ghazi A., Demont-Caulet N., Richarme G., Caldas T. (1999). Thermoprotection by glycine betaine and choline. Microbiology.

[55] Holtmann G., Bremer E. (2004). Thermoprotection of Bacillus subtilis by exogenously provided glycine betaine and structurally related compatible solutes: Involvement of Opu transporters. J. Bacteriol..

[56] Singer M.A., Lindquist S. (1998). Thermotolerance in *Saccharomyces cerevisiae*: The Yin and Yang of trehalose. Trends Biotechnol..

[57] Hottiger T., Schmutz P., Wiemken A. (1987). Heat-induced accumulation and futile cycling of trehalose in *Saccharomyces cerevisiae*. J. Bacteriol..

[58] Hazell B., Nevalainen H., Attfield P. (1995). Evidence that the *Saccharomyces cerevisiae CIF1 (GGS1/TPS1)* gene modulates heat shock response positively. FEBS Lett..

[59] Fitz A. (1878). Ueber Spaltpilzgährungen. Ber. Dtsch. Chem. Ges..

