# Supplementary material for: Taking Advantage of Bacterial Adaptation in Order to Optimize Industrial Production of Dry Propionibacterium freudenreichii
Source: Microorganisms. 2019 Oct 22;7(10):477. doi: 10.3390/microorganisms7100477 (PMC6843336; doi:10.3390/microorganisms7100477)
Supplement: Supplementary file 1 [file microorganisms-07-00477-s001.zip › microorganisms-613877-supplementary.pptx]

## Slide 1
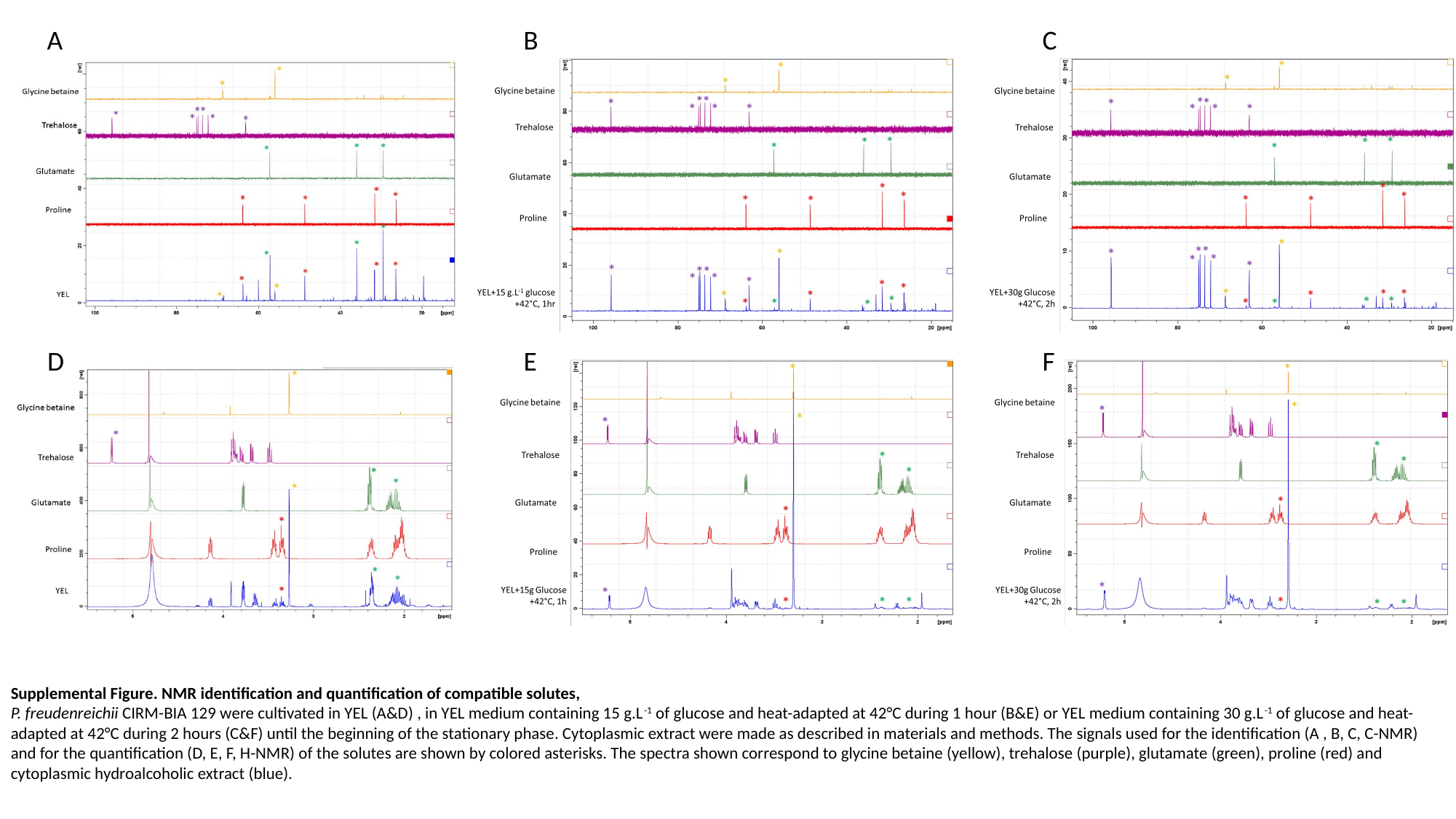

A
B
C
D
E
F
Supplemental Figure. NMR identification and quantification of compatible solutes,
P. freudenreichii CIRM-BIA 129 were cultivated in YEL (A&D) , in YEL medium containing 15 g.L-1 of glucose and heat-adapted at 42°C during 1 hour (B&E) or YEL medium containing 30 g.L-1 of glucose and heat-adapted at 42°C during 2 hours (C&F) until the beginning of the stationary phase. Cytoplasmic extract were made as described in materials and methods. The signals used for the identification (A , B, C, C-NMR) and for the quantification (D, E, F, H-NMR) of the solutes are shown by colored asterisks. The spectra shown correspond to glycine betaine (yellow), trehalose (purple), glutamate (green), proline (red) and cytoplasmic hydroalcoholic extract (blue).
